# Where You Look Is What You Get: Individual Fixation Height Predicts Biases in Face Perception

**DOI:** 10.1162/OPMI.a.353

**Published:** 2026-05-29

**Authors:** Zeynep Ceyda Demirkan, Maximilian D. Broda, Benjamin de Haas

**Affiliations:** Department of Experimental Psychology, Justus Liebig University Giessen, Giessen, Germany; Chair of Cognitive Neuroscience, Institute of Psychology, University of Regensburg, Regensburg, Germany; Center for Mind, Brain and Behaviour (CMBB), University of Marburg and Justus Liebig University Giessen, Marburg and Giessen, Germany

**Keywords:** individual differences, eye movements, face perception

## Abstract

Individuals show robust differences in where they look on a face. Some fixate closer to the eyes while others fixate closer to the mouth. While humans infer mental states and traits from specific facial regions and their expressions, such idiosyncratic gaze patterns may shape how individual features contribute to the overall impression of a face. We hypothesized that an individual’s habitual fixation behavior biases their impression of a face to be aligned more closely with the region they most often foveate. To test this idea, we analyzed fixation patterns on faces in a scene viewing task and independent perceptual judgments regarding valence, arousal, trustworthiness, dominance, and attractiveness for whole faces and face parts. A linear mixed-effects model confirmed that habitual fixation proximity to eyes, nose, and mouth explained individual differences in how well ratings for whole faces were aligned with those for the corresponding isolated feature. Building on this finding, we ran a second experiment to test whether individual differences in face fixations can predict diverging impressions of trustworthiness in appropriately manipulated images. Observers reported which of two consecutively presented versions of a subtly manipulated face they perceived to be more trustworthy. Consistent with our hypothesis, individuals who tend to fixate lower down or higher up were weakly biased to prefer versions of the face with a more trustworthy bottom or top, respectively. Together, our findings indicate that individual differences in gaze can systematically bias our sometimes-diverging impression of a face.

## INTRODUCTION

Face perception is fundamental to human social interactions, enabling individuals to recognize others, infer their emotional state (Adolphs, 2002; Horstmann, 2003), and rapidly form impressions about personality traits such as trustworthiness, dominance, and attractiveness (Oosterhof & Todorov, 2008; Todorov, 2017). These impressions can shape social decisions, from choosing whom to approach to whom we elect to positions of power (Oosterhof & Todorov, 2008). Understanding how these impressions are formed is therefore central to research in social vision.

One major factor guiding face perception is where we look. Generally, eye and mouth processing seem tuned to their typical locations in the upper and lower parafovea, respectively (de Haas & Schwarzkopf, 2018; de Haas et al., 2016, 2021). When a saccade is initiated outside the face, observers tend to land near the vertical midline slightly below the eyes (Hsiao & Cottrell, 2008), a pattern consistent with predictions from a symmetrically foveated Bayesian ideal observer model, which places the optimal fixation location just below the eyes for identity recognition (Peterson & Eckstein, 2012). When it comes to facial expressions, however, decades of work on diagnostic regions have shown that the upper and lower face differentially contribute to recognizing emotional expressions. Gaze typically concentrates on the eyes and brows when decoding fear, sadness (Wegrzyn et al., 2017), or anger (Schurgin et al., 2014), whereas happiness and disgust are more strongly associated with looking at the mouth region (Eisenbarth & Alpers, 2011; Schurgin et al., 2014; Wegrzyn et al., 2017). These findings suggest a tight relationship between where observers look and what they perceive in a face.

Interestingly, a parallel strand of research uncovered stable individual differences in face-directed gaze, which generalize from screen-based viewing of static images (Broda & de Haas, 2022a; Peterson & Eckstein, 2013) and videos (Broda & de Haas, 2022b) to real-world interactions (Peterson et al., 2016). Some observers on average fixate the eye region, while others tend to fixate lower down, e.g., on the nose or even mouth region (Peterson & Eckstein, 2012, 2013). The causes and consequences of these individual differences are largely unclear. While ‘eye avoidance’ has been discussed in the context of social cognition, anxiety and autism (Frazier et al., 2017; Klin et al., 2002; Orban de Xivry et al., 2008; Tanaka & Sung, 2016), more recent work has shown that spatial biases in face fixations generalize to inanimate objects and thus appear domain general (Broda & de Haas, 2024). And despite ideal observer predictions (Peterson & Eckstein, 2012), individuals that tend to fixate lower in a face do not benefit from forced fixations just below the eye region (Peterson & Eckstein, 2013).

However, given that different regions of a face are more or less diagnostic of different facial expressions (see above), individual differences in gaze may have consequences for the perception of facial traits and states. Different observers may extract different information from the same face. Specifically, the impression of a face may be biased towards the region which a given individual tends to foveate habitually. Here, we test this hypothesis in two steps, building on the previous finding that observers readily infer valence, arousal, trustworthiness, and dominance from isolated facial features as well as whole faces (Broda & de Haas, 2023).

In our first study, participants rated isolated facial features (eyes, mouths) and then corresponding whole faces on multiple dimensions (valence, arousal, trustworthiness, dominance, attractiveness), which allowed us to determine how well their whole-face ratings were aligned with either feature in isolation. In addition, we measured their individual face fixation preferences in an independent free-viewing experiment, using complex scenes. In line with our hypothesis, we found that the degree to which ratings for either facial feature predicted whole-face ratings declined with participants’ habitual fixation distance from that feature. In a second, two-alternatives forced choice experiment we concentrated on perceived trustworthiness and used composite face chimaeras combining subtle, but conflicting cues (e.g., a slightly trustworthy top with a neutral bottom and vice versa). Our aim was to test whether individual fixation biases can lead to diverging preferences when it comes to selecting the more trustworthy out of two candidate faces. The likelihood of choosing the more trustworthy face according to a given feature indeed trended to decline with habitual fixation distance from that feature. Taken together, our results show that individual gaze can shape individual face perception.

## METHODS

### Participants

In Experiment 1, sixty-two healthy adults (*N* = 62; 38 females; mean age = 24 years, *SD* = 3.8) with normal or corrected-to-normal vision participated. Fourteen participants had previously completed the free-viewing task using the same stimuli (de Haas et al., 2019) and were invited to take part in the online survey. The remaining participants completed both the survey and the eye-tracking session sequentially in the lab (total duration ≈ 2.5 h). Participants received either course credit (1 credit/h) or monetary compensation (€10/h; online-only participants: €4). All participants were proficient in German.

In Experiment 2, seventy-three participants were recruited; data from three were excluded due to eye-tracking failure, resulting in a final sample of 70 participants (*N* = 70; 54 females; mean age = 24.3 years, *SD* = 4.2). All participants first completed the free-viewing task from Experiment 1, followed by a two-interval forced-choice (2-IFC) task using face chimaera stimuli (see Procedure).

Sample sizes were chosen to balance statistical power for medium effect sizes with practical constraints and are in line with previous work on individual differences in eye-movement patterns (Broda & de Haas, 2023, 2024; de Haas et al., 2019; Peterson & Eckstein, 2012, 2013). The study was approved by the local ethics committee of Justus Liebig University Giessen (Fachbereich 06) and conducted in accordance with the Declaration of Helsinki. Written informed consent was obtained from all participants.

### Stimuli

In Experiment 1, participants viewed 700 naturalistic scene images collated by Xu et al. (2014). These images depict complex everyday scenes with annotated objects and faces and are available from a public database (https://www-users.cse.umn.edu/∼qzhao/predicting.html). For the online face-rating survey, we used 30 frontal face images sourced from the fLoc localizer package (Stigliani et al., 2015) and additional frontal images sourced online to include subtle negative expressions. Each face was presented both as a full image and segmented into isolated eye, nose, and mouth regions following the procedure described by Broda and de Haas (2023).

**Figure 1.**
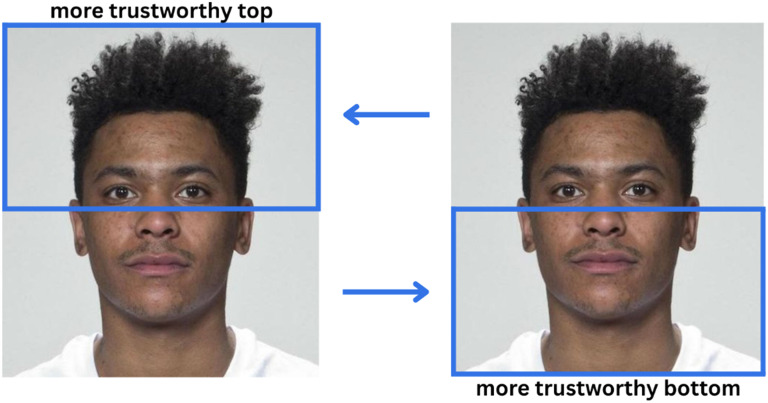
Example of face chimaera stimuli created by combining the upper-half (left) or lower-half (right) trustworthiness manipulation with the neutral face of the same identity. Blue boxes are shown to illustrate the manipulation and were not part of the stimuli as seen by participants. Stimuli were created from the images provided by Oh et al. (2020) and DeBruine and Jones (2017).

In Experiment 2, participants viewed the same 700-scene image set in the free-viewing task, followed by a two-interval forced-choice (2-IFC) task in which they judged which of two sequentially presented face chimaeras appeared more trustworthy (Figure 1). Chimaeras were created from the stimuli in Study 3b by Oh et al. (2020), which were based on 50 identities (25 female, 25 male) from the Face Research Lab London Face Set (DeBruine & Jones, 2017). We combined neutral versions of the faces (manipulation level 4: 0%) with slightly trustworthiness-enhanced versions (manipulation level 5: 13.33%). Each of the 50 faces appeared in two versions, with either the upper or lower half manipulated to appear more trustworthy, resulting in 100 unique stimuli. For this experiment we focused on subtle manipulations of trustworthiness, because this manipulation lent itself best to well blending chimaeras without conspicuous borders between face-halves (Figure 1).

### Procedure

**Figure 2.**
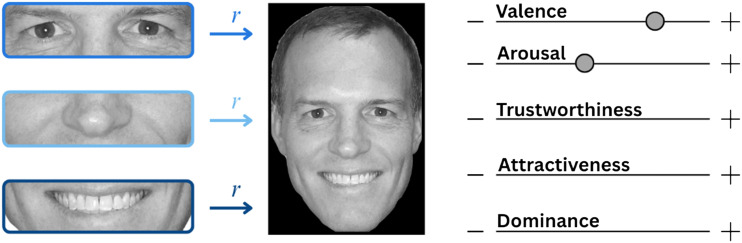
Participants were presented with isolated facial features (eyes, nose, mouth) or whole-face images and rated each stimulus on five dimensions: valence, arousal, trustworthiness, attractiveness, and dominance using continuous scales. Colored arrows indicate the correlations (*r*) between isolated-features and whole-face ratings which were calculated individually for each participant’s data. Face images were sourced from the fLoc stimulus set (Stigliani et al., 2015; including the example shown) and other online sources.

Experiment 1 consisted of two parts: an online rating survey and a free-viewing eye-tracking task. In the survey, participants rated isolated facial features (eyes, nose, mouth) and whole-face images on five dimensions—valence, arousal, trustworthiness, attractiveness, and dominance—using continuous rating scales (Figure 2). Following the procedure described by Broda and de Haas (2023), the stimulus set was shuffled once prior to the experiment, and all participants viewed the stimuli in this same fixed order. Isolated facial features (eyes, noses and mouths) were intermixed within the shuffled stimulus list and always presented before their corresponding whole-face images. Participants completed the survey on their own devices; therefore, screen resolution, viewing distance, and stimulus size in degrees of visual angle could not be controlled.

In the free-viewing task, participants were seated with their heads stabilized using a chin-and-forehead rest, positioned 55 cm from the display screen. Stimuli were presented on a 526 × 296 mm display (3,840 × 2,160 pixels) at a size of 2,400 × 1,800 pixels, corresponding to 33.28 × 25.27 degrees of visual angle. Eye movements were recorded at 1 kHz using an EyeLink 1000 tower-mount system (SR Research). Stimuli were presented in blocks of 100, each preceded by a nine-point calibration and validation procedure. Participants initiated each trial by fixating a central disk and pressing a button. Each image was then displayed for 3 s, during which participants could freely view the scene. Image order was identical across participants.

Experiment 2 followed the same procedure for the free-viewing task and added a two-interval forced-choice (2-IFC) task using the face chimaera stimuli. For the 2-IFC chimaera task, stimuli were presented at 1,000 × 1,118 pixels, corresponding to 14.20 × 15.86 degrees of visual angle on a display with a resolution of 3,840 × 2,160 pixels (526 × 296 mm display), viewed from 55 cm. Each trial began with a fixation dot presented either 15 degrees to the left or right along the horizontal midline (counterbalanced across trials). This way, participants had to saccade into the face presented subsequently and we minimized the risk of biasing their vertical landing position. Participants pressed the space bar when ready, after which two versions of the same face were presented centrally, sequentially for 500 ms each, separated by a 500 ms interstimulus interval (ISI). In half the trials the face with the trustworthy upper-half appeared first (pseudorandom order). Participants indicated which face appeared more trustworthy via button press. Each of the 50 faces was presented once per condition, resulting in 100 trials in total.

### Analyses

For both experiments, fixations during the free-viewing task were extracted using the Eyelink parser and subsequently analyzed in MATLAB R2022b (MathWorks, Natick, MA, USA). Fixations were included if their onset latency exceeded 100 ms and their duration exceeded 100 ms. Vertical fixation position was normalized within each face using the eye–mouth distance, with fixations at the mouth defined as 0 and fixations at the eyes defined as 1 yielding a relative fixation height (*γ*) (cf. Peterson et al., 2016). Eye and mouth coordinates were obtained from the facial feature masks provided by Broda and de Haas (2022a). This also allowed us to compute the distance to either of these features and to the nose region, which was coded as a height of 0.40 relative to the mouth-eyes distance (i.e., 40% of the distance from the mouth toward the eyes). The average fixation height and distance to each feature across all face fixations were computed per participant and used as predictors in subsequent analyses. To test the robustness of individual differences in face fixation height, we calculated the split-half consistency of average face fixation heights across odd and even images. Additionally, we examined whether participants’ fixation heights on faces correlated with their fixation heights on inanimate objects, as previously reported by Broda and de Haas (2024).

For Experiment 1, fixation data were combined with survey-based ratings. Participants rated isolated facial features (eyes, nose, mouth) and whole faces on five dimensions: valence, arousal, trustworthiness, attractiveness, and dominance. For each participant, correlations were computed between feature-specific ratings and corresponding whole-face ratings. These correlations served as dependent variables in linear mixed-effects models (LME) implemented in MATLAB (fitlme, Statistics and Machine Learning Toolbox). Fixed effects predictors included combinations of fixation distance (continuous), feature (categorical), and rating dimension (categorical), with random intercepts for each subject to account for individual variability. Comparisons across models including different subsets of these predictors were performed using Akaike Information Criterion (AIC; Akaike, 1974) and Bayesian Information Criterion (BIC; Schwarz, 1978). For Experiment 2, choice data from the 2-IFC chimaera task were summarized as the proportion of choices selecting the chimaera with the more trustworthy bottom-half as more trustworthy. Internal consistency of individual differences in this bottom-choice bias was assessed via split-half correlations. To test whether gaze biases predicted choice bias, we correlated individual differences in mean relative fixation height during free viewing with the bottom-choice bias.

### Statistics

To evaluate the robustness of the linear mixed-effects (LME) model in Experiment 1, model assumptions were assessed using residual diagnostics. Normality of residuals was evaluated using a histogram, quantile–quantile (Q–Q) plot and Kolmogorov–Smirnov test. Homoscedasticity was assessed by visual inspection of residuals versus fitted values plots. Because correlation coefficients were used as the dependent variable (FullFaceCorr), Fisher’s *Z* transformation was applied prior to analysis to improve normality and variance stability. Multicollinearity among fixed effects was assessed using Variance Inflation Factors (VIF).

To further assess the robustness of the correlation observed in Experiment 2, we computed non-parametric bootstrap confidence intervals (95%, 10,000 resamples). In addition, Bayesian correlation analysis was performed to quantify evidence for the alternative hypothesis using the JZS Bayes Factor (BF_10_). To evaluate robustness to potential outliers, we additionally calculated the 20% percentage bend correlation, which reduces the influence of extreme observations.

## RESULTS

### Experiment 1

Participants showed reliable individual differences in their average fixation height within faces (split half consistency *r* = .884, *p* < .001), which generalized to average fixation height within inanimate objects (*r* = .815, *p* < .001, replicating Broda & de Haas, 2024).

The results of the LME model are displayed in Table 1. As hypothesized, individual fixation distance from a feature during the free viewing experiment (FixDist) was a significant negative predictor for the correlation between feature-specific and whole-face ratings in the online survey (*t*(922) = −2.64, *p* = .008; highlighted in red in Table 1). This indicates that participants who fixate more closely to a specific feature tend to weigh that feature more strongly when forming whole-face judgments.

**Table 1.**
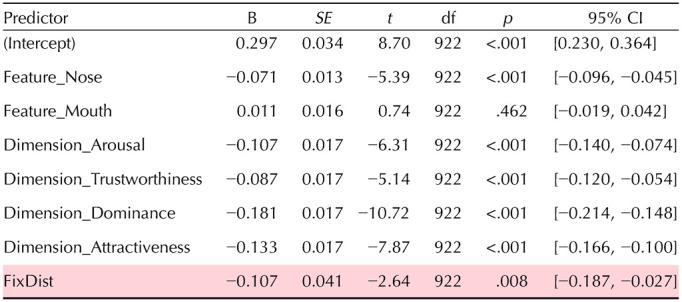
Results of the linear mixed effects model of alignment between ratings of isolated features and whole faces.

*Notes*. Fixed effects (fixation distance, features, rating dimensions) predicting the feature − whole face rating correlations using a linear mixed effects model. The effect of fixation distance is highlighted in red. All fixed effects of features and dimensions are relative to the reference baseline Feature: Eyes and Dimension: Valence.

The model was specified as:

$$\text{FullFaceCorr}∼\text{FixDist}+\text{Feature}+\text{Dimension}+\left(1|\text{Subject}\right)$$

The model also yielded a fixed intercept of 0.297 (*t*(922) = 8.70, *p* < .001), reflecting the average baseline correlation between part-based and whole-face ratings when predictors are at their reference levels (Feature: Eye and Dimension: Valence). Fixed Feature and Dimension effects indicated that, overall, feature-whole face correlations were stronger for Valence compared to all other dimensions (all *p* < .001); and compared to Eyes, Nose, but not Mouth ratings correlated significantly weaker with those for whole faces (*t*(922) = −5.39, *p* < .001). These results are broadly in line with the pattern reported by Broda and de Haas (2023). Finally, a random intercept for Subject (*SD* = 0.062, 95% CI [0.048, 0.080]) captured reliable individual differences in the overall consistency between feature-based and whole-face ratings.

Taken together, habitual fixation distance from a given feature was a significant predictor of how strongly individual whole face ratings were aligned with that feature.

To evaluate model fit, we compared the primary model to an expanded model including feature × dimension interactions. Although the expanded model yielded a slightly lower AIC (maximum ΔAIC = −7.37), it produced a higher BIC (maximum ΔBIC = +31.31), reflecting increased model complexity. Because the interaction terms did not alter the pattern of main effects and were not theoretically central, we report the more parsimonious primary model.

Residual diagnostics indicated that model assumptions were adequately met. Residuals were well fit by a normal distribution, as indicated by their histogram and Q–Q plot, and the Kolmogorov–Smirnov test did not indicate a deviation from normality (*p* = .487). Visual inspection of residuals versus fitted values revealed no systematic heteroscedasticity. Variance Inflation Factors were low for all predictors (range = 1.337–1.694), well below commonly accepted thresholds, indicating independence among fixed effects.

To assess potential response bias, the proportion of extreme ratings (0% or 100%) was calculated for each participant, feature, and rating dimension. Extreme responses were generally infrequent (mean = 0.70%, max = 46.67%) and showed no systematic concentration in specific conditions, indicating that the observed effects were not driven by habitual use of scale endpoints.

### Experiment 2

Participants again showed reliable individual differences in their average fixation height within faces (split half consistency *r* = .96, *p* < .001), which also generalized to average fixation height within inanimate objects (*r* = .77, *p* < .001, replicating Broda & de Haas, 2024).

**Figure 3.**
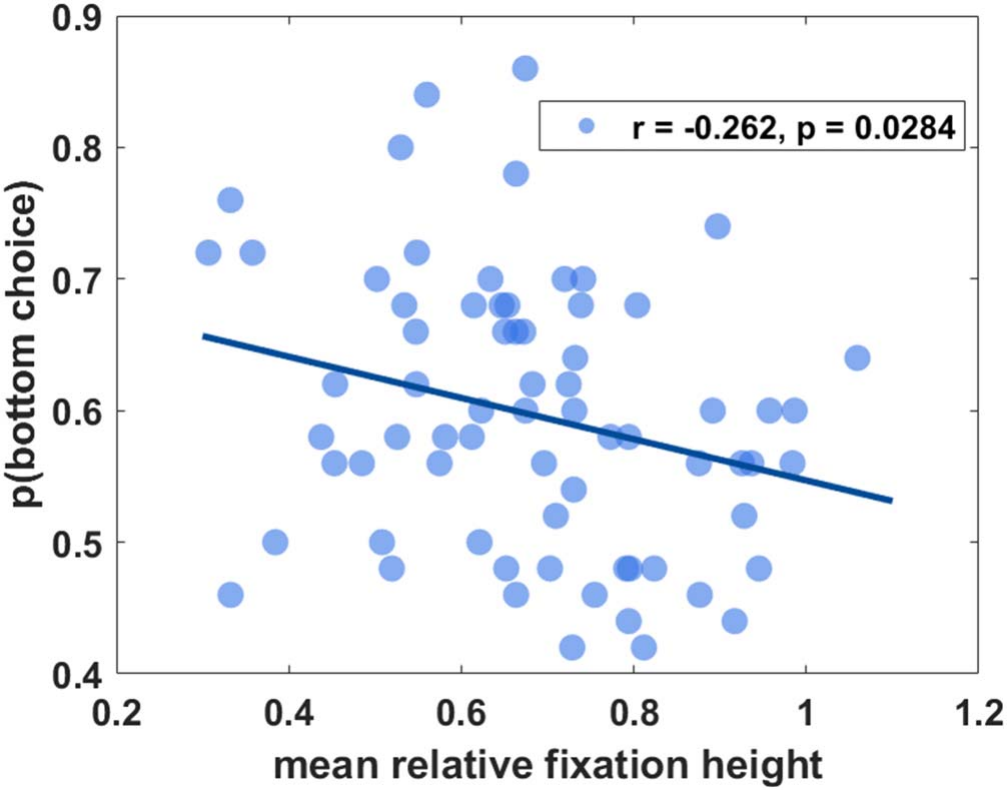
Correlation between mean relative fixation height in faces and bottom choice probability. Each point represents data from one participant. The *x* axis indicates the participants’ mean relative fixation height in faces during the free viewing task, and the *y* axis indicates the proportion of trustworthiness preference choices for faces with a trustworthy bottom over faces with a trustworthy top during the two-interval forced-choice task. The solid line depicts the least-squares regression fit.

We now examined whether participants’ fixation height predicted their behavioral choices in a 2-IFC face chimera task. Across participants (*N* = 70), the mean relative fixation height was significantly negatively correlated with the proportion of choices of the face with a more trustworthy bottom-half (*r* = −.262, *p* = .0284; Figure 3). This negative relationship remained robust against outliers and distributional assumptions (bootstrap 95% CI [−0.461, −0.048]; Percentage Bend *r*_*pb*_ = −.271, *p* = .0231). However, a Bayesian analysis using a default JZS prior yielded only anecdotal evidence for the effect (BF_10_ = 1.025). Although the effect sizes were modest, this indicates that participants who tended to fixate lower on faces were also somewhat more likely to judge the bottom-half manipulated versions as more trustworthy.

## DISCUSSION

We asked whether where people tend to look on a face determines how strongly individual features shape their impression of the whole face. In the first experiment, we found that participants who tend to fixate closer to a given facial feature showed stronger alignment between their ratings of the whole face and their ratings of that feature. In a second experiment, fixation height was statistically associated with behavioral choices in a face-chimaera trustworthiness task: The lower a given individual tends to fixate, the more likely they were to prefer a face with a trustworthy lower half over a face with a trustworthy top. However, a Bayesian analysis with a JZS prior suggested that the evidential weight of this second finding was minimal. Even though the effect size in the second study was small, the findings of both studies converge to suggest that individual fixation tendencies shape face perception.

The trustworthiness manipulations in the second study were deliberately subtle. Stronger manipulations of trustworthiness are associated with changes in facial width which would have produced salient contour misalignments between top and bottom halves. Future studies may be able utilize stronger manipulations that can be integrated into a single face, such as varying facial expressions. Such an approach may yield stronger evidence regarding the hypothesized gaze-dependent divergence of facial impressions.

Our findings replicate prior reports of stable vertical fixation differences across static, dynamic, and real-world contexts (Broda & de Haas, 2022a, 2022b; Peterson & Eckstein, 2013; Peterson et al., 2016) and show that they have measurable perceptual consequences. Impressions of a face are biased towards those features a given individual tends to foveate. We also replicated the strong cross-domain relationship between fixation heights for faces and objects reported by Broda and de Haas (2024) in our data (*r* = .82 and *r* = .77 in Experiments 1 and 2, respectively), reinforcing the idea that fixation preferences reflect domain-general aspects of active vision rather than face-specific strategies. This suggests that domain-general visual exploration habits are linked to how we interpret complex, socially relevant stimuli. However, neither our data nor that of Broda and de Haas (2024) reveal the developmental origins of this association. One possibility is that domain-general fixation biases are down to low-level anisotropies of the individual visual system and ultimately modulate face perception. Alternatively, it is conceivable that factors related to social cognition or experience lead to e.g., eye avoidance (Frazier et al., 2017; Klin et al., 2002; Orban de Xivry et al., 2008; Tanaka & Sung, 2016) and over time, these fixation biases generalize to objects and perhaps even shape the development of visual field biases. Longitudinal or training studies could test whether modifying gaze behavior systematically alters how facial information is integrated into social judgments. Similarly, neuroimaging work could probe whether individual differences in gaze go along with distinct representational geometries within face-responsive cortical regions or altered correlations between retinotopic and face part tuning (de Haas & Schwarzkopf, 2018; de Haas et al., 2016, 2021; Henriksson et al., 2015).

The importance of face perception for social cognition also raises the question of downstream effects of the perceptual modulations we found. One important question is how individual fixation biases relate to emotion recognition performance. In our experiments there were no pre-defined ‘correct’ answers. Participants chose between ambiguous pairs of faces or provided gradual ratings for faces and features without pre-defined expressions. However, previous studies have shown that performance in categorizing predefined (extreme) facial expressions is optimal when sampling the features which are more diagnostic for a given expression (Blais et al., 2012; Eisenbarth & Alpers, 2011; Kim et al., 2022; Schurgin et al., 2014; Wegrzyn et al., 2017). This raises the possibility that individual fixation tendencies interact with the sensitivity for recognizing certain facial expressions, a hypothesis that could be tested in future experiments.

In summary, our findings underscore the importance of gaze and the individual observer for social vision. What we see depends on who is looking, and where. Understanding how idiosyncratic fixation strategies shape perception may ultimately help explain the remarkable diversity of social impressions humans can form from the same visual input.

## CONCLUSION

Across two experiments, we found that stable individual differences in vertical fixation position tend to bias how facial features are weighted when forming impressions of faces. Observers who preferentially fixate higher on the face rely more strongly on upper-face information, whereas those who fixate lower weigh information from lower-face more heavily. These differences not only modulate the alignment between feature and whole-face judgments but are also weakly correlated with divergent choices in a subtle trustworthiness discrimination task. Together, our findings suggest that idiosyncratic gaze shapes individual face perception, linking habitual sampling behavior to variability in face impressions.

## ACKNOWLEDGMENTS

Large language models (OpenAI’s ChatGPT, Google’s Gemini, Anthropic’s Claude) were used to suggest language improvements for this manuscript, as well as improvements for the underlying MATLAB code. The authors take full responsibility for the integrity and accuracy of all analyses and of the final manuscript.

## FUNDING INFORMATION

This work was supported by the Deutsche Forschungsgemeinschaft (German Research Foundation, DFG) under Germany’s Excellence Strategy (EXC 3066/1 “The Adaptive Mind”, Project No. 533717223) and Project No. 222641018-SFB/TRR 135 TP C9, as well as ERC Starting Grant No. 852885 INDIVISUAL.

## AUTHOR CONTRIBUTIONS

Z.C.D.: Conceptualization; Data curation; Formal analysis; Investigation; Methodology; Writing – original draft. M.D.B.: Conceptualization; Writing – review & editing; B.d.H.: Conceptualization; Formal analysis; Methodology; Writing – review & editing.

## DATA AND CODE AVAILABILITY STATEMENT

The data and analyses code are available at https://osf.io/h5gfk.

## References

[bib1] Adolphs, R. (2002). Recognizing emotion from facial expressions: Psychological and neurological mechanisms. Behavioral and Cognitive Neuroscience Reviews, 1(1), 21–62. 10.1177/1534582302001001003, 17715585

[bib2] Akaike, H. (1974). A new look at the statistical model identification. IEEE Transactions on Automatic Control, 19(6), 716–723. 10.1109/TAC.1974.1100705

[bib3] Blais, C., Roy, C., Fiset, D., Arguin, M., & Gosselin, F. (2012). The eyes are not the window to basic emotions. Neuropsychologia, 50(12), 2830–2838. 10.1016/j.neuropsychologia.2012.08.010, 22974675

[bib4] Broda, M. D., & de Haas, B. (2022a). Individual differences in looking at persons in scenes. Journal of Vision, 22(12), 9. 10.1167/jov.22.12.9, 36342691 PMC9652713

[bib5] Broda, M. D., & de Haas, B. (2022b). Individual fixation tendencies in person viewing generalize from images to videos. i-Perception, 13(6), 20416695221128844. 10.1177/20416695221128844, 36353505 PMC9638695

[bib6] Broda, M. D., & de Haas, B. (2023). Reading the mind in the nose. i-Perception, 14(2), 20416695231163449. 10.1177/20416695231163449, 36960407 PMC10028657

[bib7] Broda, M. D., & de Haas, B. (2024). Individual differences in human gaze behavior generalize from faces to objects. Proceedings of the National Academy of Sciences, 121(12), e2322149121. 10.1073/pnas.2322149121, 38470925 PMC10963009

[bib8] de Haas, B., Iakovidis, A. L., Schwarzkopf, D. S., & Gegenfurtner, K. R. (2019). Individual differences in visual salience vary along semantic dimensions. Proceedings of the National Academy of Sciences, 116(24), 11687–11692. 10.1073/pnas.1820553116, 31138705 PMC6576124

[bib9] de Haas, B., & Schwarzkopf, D. S. (2018). Feature-location effects in the Thatcher illusion. Journal of Vision, 18(4), 16. 10.1167/18.4.16, 29710306

[bib10] de Haas, B., Schwarzkopf, D. S., Alvarez, I., Lawson, R. P., Henriksson, L., Kriegeskorte, N., & Rees, G. (2016). Perception and processing of faces in the human brain is tuned to typical feature locations. Journal of Neuroscience, 36(36), 9289–9302. 10.1523/JNEUROSCI.4131-14.2016, 27605606 PMC5013182

[bib11] de Haas, B., Sereno, M. I., & Schwarzkopf, D. S. (2021). Inferior occipital gyrus is organized along common gradients of spatial and face-part selectivity. Journal of Neuroscience, 41(25), 5511–5521. 10.1523/JNEUROSCI.2415-20.2021, 34016715 PMC8221599

[bib12] DeBruine, L., & Jones, B. (2017). Face Research Lab London Set. figshare. 10.6084/m9.figshare.5047666

[bib13] Eisenbarth, H., & Alpers, G. W. (2011). Happy mouth and sad eyes: Scanning emotional facial expressions. Emotion, 11(4), 860–865. 10.1037/a0022758, 21859204

[bib15] Frazier, T. W., Strauss, M., Klingemier, E. W., Zetzer, E. E., Hardan, A. Y., Eng, C., & Youngstrom, E. A. (2017). A meta-analysis of gaze differences to social and nonsocial information between individuals with and without autism. Journal of the American Academy of Child & Adolescent Psychiatry, 56(7), 546–555. 10.1016/j.jaac.2017.05.005, 28647006 PMC5578719

[bib14] Henriksson, L., Mur, M., & Kriegeskorte, N. (2015). Faciotopy—A face-feature map with face-like topology in the human occipital face area. Cortex, 72, 156–167. 10.1016/j.cortex.2015.06.030, 26235800 PMC4643680

[bib16] Horstmann, G. (2003). What do facial expressions convey: Feeling states, behavioral intentions, or actions requests? Emotion, 3(2), 150–166. 10.1037/1528-3542.3.2.150, 12899416

[bib17] Hsiao, J. H., & Cottrell, G. (2008). Two fixations suffice in face recognition. Psychological Science, 19(10), 998–1006. 10.1111/j.1467-9280.2008.02191.x, 19000210 PMC7360057

[bib18] Kim, M., Cho, Y., & Kim, S.-Y. (2022). Effects of diagnostic regions on facial emotion recognition: The Moving Window Technique. Frontiers in Psychology, 13, 966623. 10.3389/fpsyg.2022.966623, 36186300 PMC9518794

[bib19] Klin, A., Jones, W., Schultz, R., Volkmar, F., & Cohen, D. (2002). Visual fixation patterns during viewing of naturalistic social situations as predictors of social competence in individuals with autism. Archives of General Psychiatry, 59(9), 809–816. 10.1001/archpsyc.59.9.809, 12215080

[bib20] Oh, D., Dotsch, R., Porter, J., & Todorov, A. (2020). Gender biases in impressions from faces: Empirical studies and computational models. Journal of Experimental Psychology: General, 149(2), 323–342. 10.1037/xge0000638, 31294585

[bib21] Oosterhof, N. N., & Todorov, A. (2008). The functional basis of face evaluation. Proceedings of the National Academy of Sciences, 105(32), 11087–11092. 10.1073/pnas.0805664105, 18685089 PMC2516255

[bib22] Orban de Xivry, J.-J., Ramon, M., Lefèvre, P., & Rossion, B. (2008). Reduced fixation on the upper area of personally familiar faces following acquired prosopagnosia. Journal of Neuropsychology, 2(1), 245–268. 10.1348/174866407x260199, 19334313

[bib23] Peterson, M. F., & Eckstein, M. P. (2012). Looking just below the eyes is optimal across face recognition tasks. Proceedings of the National Academy of Sciences, 109(48), E3314–E3323. 10.1073/pnas.1214269109, 23150543 PMC3511732

[bib24] Peterson, M. F., & Eckstein, M. P. (2013). Individual differences in eye movements during face identification reflect observer-specific optimal points of fixation. Psychological Science, 24(7), 1216–1225. 10.1177/0956797612471684, 23740552 PMC6590077

[bib25] Peterson, M. F., Lin, J., Zaun, I., & Kanwisher, N. (2016). Individual differences in face-looking behavior generalize from the lab to the world. Journal of Vision, 16(7), 12. 10.1167/16.7.12, 27191940

[bib26] Schurgin, M. W., Nelson, J., Iida, S., Ohira, H., Chiao, J. Y., & Franconeri, S. L. (2014). Eye movements during emotion recognition in faces. Journal of Vision, 14(13), 14. 10.1167/14.13.14, 25406159

[bib27] Schwarz, G. (1978). Estimating the dimension of a model. The Annals of Statistics, 6(2), 461–464. 10.1214/aos/1176344136

[bib28] Stigliani, A., Weiner, K. S., & Grill-Spector, K. (2015). Temporal processing capacity in high-level visual cortex is domain specific. Journal of Neuroscience, 35(36), 12412–12424. 10.1523/JNEUROSCI.4822-14.2015, 26354910 PMC4563034

[bib29] Tanaka, J. W., & Sung, A. (2016). The “eye avoidance” hypothesis of autism face processing. Journal of Autism and Developmental Disorders, 46(5), 1538–1552. 10.1007/s10803-013-1976-7, 24150885 PMC3997654

[bib32] Todorov, A. (2017). Face value: The irresistible influence of first impressions. Princeton University Press. 10.1515/9781400885725

[bib33] Wegrzyn, M., Vogt, M., Kireclioglu, B., Schneider, J., & Kissler, J. (2017). Mapping the emotional face. How individual face parts contribute to successful emotion recognition. PLoS One, 12(5), e0177239. 10.1371/journal.pone.0177239, 28493921 PMC5426715

[bib34] Xu, J., Jiang, M., Wang, S., Kankanhalli, M. S., & Zhao, Q. (2014). Predicting human gaze beyond pixels. Journal of Vision, 14(1), 28. 10.1167/14.1.28, 24474825

